# The thermal dependence and molecular basis of physiological color change in *Takydromus septentrionalis* (Lacertidae)

**DOI:** 10.1242/bio.058503

**Published:** 2021-03-26

**Authors:** Kun Guo, Jun Zhong, Lin Zhu, Fan Xie, Yu Du, Xiang Ji

**Affiliations:** 1 Jiangsu Key Laboratory for Biodiversity and Biotechnology, College of Life Sciences, Nanjing Normal University, Nanjing 210023, Jiangsu, China; 2 Institute of Biodiversity Conservation and Utilization, College of Life and Environmental Sciences, Wenzhou University, Wenzhou 325035, Zhejiang, China; 3 MOE Key Laboratory of Utilization and Conservation for Tropical Marine Bioresources, Hainan Tropical Ocean University, Sanya 572022, Hainan, China

**Keywords:** Cytoskeleton, Motor protein, Physiological color change, RNA sequencing, Thermoregulation

## Abstract

One of the main functions of physiological color change is thermoregulation. This change occurs much more rapidly than morphological color change, but the underlying mechanism remains poorly understood. Here, we studied the thermal dependence and molecular basis of physiological color change in lizards using *Takydromus septentrionalis* (Lacertidae) as the model system. Body color was thermally sensitive, becoming increasingly light as body temperatures deviated from the level (∼30°C) preferred by this species. We identified 3389 differentially expressed genes (DEGs) between lizards at 24°C and 30°C, and 1,097 DEGs between lizards at 36°C and 30°C. Temperature affected the cAMP signal pathway, motor proteins, cytoskeleton, and the expression of genes related to melanocyte-stimulating hormone (MSH) and melanocyte-concentrating hormone (MCH). Our data suggest that the role of physiological color change in thermoregulation is achieved in *T. septentrionalis* by altering the arrangement of pigments and thus the amount of solar radiation absorbed and reflected. G protein-coupling system inhibits adenylate cyclase activity to transform ATP into cAMP and thereby causes rapid pigment aggregation. MCH deactivates the G proteins and thereby initiates pigment dispersion. This mechanism differs from that reported for teleost fish where MCH activates the G proteins and thereby causes pigment aggregation.

This article has an associated First Person interview with the first author of the paper.

## Abstract (precis)

**Summary:** G protein-coupling system inhibits adenylate cyclase activity to transform ATP into cAMP and thereby causes rapid pigment aggregation. MCH deactivates the G proteins and thereby initiates pigment dispersion.

## INTRODUCTION

Color change is mediated by synchronous intracellular transport of pigmented organelles in chromatophores and has functional roles in signaling (visual communication), background matching (camouflage), and thermoregulation (Baling et al., 2016; Smith et al., 2016a,b). Previous studies have focused more intensively on the former two aspects than on the latter one. Thermoregulation is important for animals because body temperature affects numerous physiological and behavioral performances (Angilletta, 2001). Animals regulate body temperature by a combination of behavioral and physiological mechanisms. Body temperature is dependent on environmental temperature in ectotherms, and this is especially true for those in thermally uniformed environments where behavioral thermoregulation is constrained (Angilletta, 2001; Muri et al., 2015). Many animals are vulnerable to climate warming primarily because the increased heat loads constrain the activities necessary to maintain a normal life (Bakken, 1992; Huey et al., 2009, 2010; Sinervo et al., 2010; Wang et al., 2017). One way of thermoregulation involves a rapid change in coloration, which alters absorption and reflection of radiant heat (Stuart-Fox and Moussalli, 2009; Smith et al., 2016b). There are two major types of color change: morphological color change occurs over days or months (Aspengren et al., 2009); physiological color change occurs much more rapidly in a few seconds or minutes (Fujii, 1993). Chromatophores originate from the embryonic nerve crest and form units of migrating dermal chromatophores on the outer layer of the epidermis (Bagnara and Hadley, 1969; Fujii, 1993). The density and aggregation of pigments in chromatophores determine skin color (Grether et al., 2004). Morphological color is determined by the number and quality of chromatophores in the dermis, and physiological color by the relative state of pigment dispersion and aggregation within each chromatophore (Ligon and McCartney, 2016).

Physiological color change occurs in response to environmental signals and is mediated by nervous-fibers or the endocrine system (Fujii, 1993; Ligon and McCartney, 2016). In fish and reptiles, the sympathetic nervous system responds to stress and releases catecholamine (Summers and Greenberg, 1994), which binds to adrenoceptors on the plasma membrane and increases the concentration of secondary messengers such as cyclic adenosine monophosphate (cAMP) (Nery and Castrucci, 1997). High concentrations of cAMP activate protein kinase-A, leading to protein phosphorylation (Nery and Castrucci, 1997). Phosphorylated proteins detach from the cytoskeleton and move along cytoskeletal filaments to the cell periphery (Nery and Castrucci, 1997). Microtubule- and microfilament-dependent pigment transportation requires the assistance of motor proteins such as kinesin, dynein and myosin (Rogers and Gelfand, 1988; Ligon and McCartney, 2016). The genes *MYO3* and *MYO5* encode classical myosin I proteins (Goodson et al., 1996). Noradrenaline, a strong pigment-aggregating hormone, can activate cAMP synthetase adenylate cyclase and stimulate melanosome diffusion in fish and amphibians (Nilsson et al., 2013). Melanocyte-stimulating hormone (MSH) and melanocyte-concentrating hormone (MCH) regulate body color change (Sugimoto, 2002; Mizusawa et al., 2013; Nilsson et al., 2013). The MSH and MCH receptors, *MC1R* and *MCHR*, both participate in color change in fish, amphibians, birds and mammals (Nilsson et al., 2013; Bertolesi and McFarlane, 2018). *Opn4x* in amphibians and the orthologous *Opn4m* in mammals regulate α-MSH levels though a neuroendocrine circuit (Bertolesi and McFarlane, 2018).

Many lizards have the ability to change body color. For example, bearded dragons (*Pogona vitticeps*) darken or lighten their color, thereby altering the amount of solar radiation absorbed or reflected (Stuart-Fox and Moussalli, 2009; Nilsson et al., 2013; Smith et al., 2016b). Solar radiation comprises ultraviolet light (UV, 290–400 nm), visible light (Vis, 400–700 nm) and near infrared light (NIR, 700–2600 nm) (Porter, 1967). Most energy exists within the Vis and NIR parts of the spectrum; however, NIR has little to no effect on camouflage because of the insensitivity of the visual system in animals to NIR (Clusella-Trullas et al., 2007). UV-Vis is the most important spectrum component affecting absorption of solar radiation in *P. vitticeps* (Smith et al., 2016b). As the dorsal surface is able to reflect light within the Vis wavelengths in lizards (Murillo et al., 2020), one may hypothesize that Vis is the most important part of solar radiation for thermoregulation. Here, we quantified dorsal coloration based on the RGB value in adult male northern grass lizards (*Takydromus septentrionalis*) and then examined the transcriptomic basis of that color change to test the hypothesis. The RGB color model is an additive color model used for numerical color specifications. The RGB value refers to luminance, which is the most important color component affecting absorption of solar radiation; the RGB value can be transformed to YCbCr color spaces, in which Y represents the luminance component (Ahirwal et al., 2007; Smith et al., 2016a).

## RESULTS

### Temperature-dependent changes in body color

**Fig. 1.**
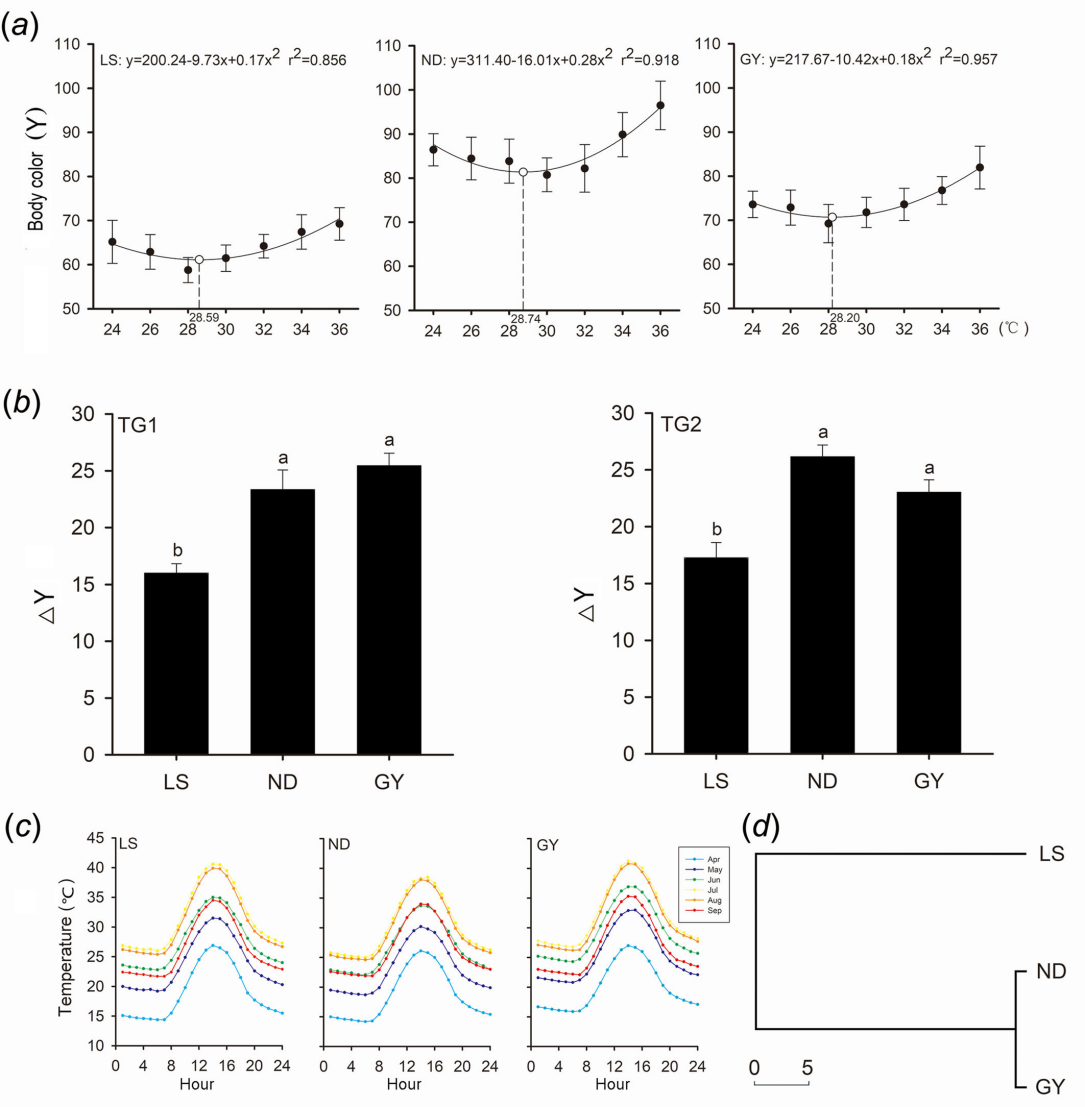
**The thermal dependence of body color change in *T. septentrionalis* from the Lishui (LS), Ningde (ND), and Guiyang (GY) populations.** (A) Functions and curves describing the relationship between body color [expressed as adjusted mean values (±s.e.) for Y, with body mass set at 6.0 g] and body temperature. (B) Mean values (+s.e.) for ΔY, the absolute difference in Y values between P1 and P2. (C) Daily mean temperatures during the most active season (from April to September) in 2013 at the three sampling localities. (D) Results of cluster analysis on 19 climate variables, showing climate differences among the three populations. TG1: a thermal gradient from 24−50°C; TG2, a thermal gradient from 12−36°C. Means with different letters differed significantly (Tukey's test, α=0.05).

Temperature affected body color luminance (*F*_6, 342_=6.76, *P*<0.001), which generally decreased with increasing temperature at lower temperatures (from 24°C to Tp) and increased with increasing temperature at higher temperatures (from Tp to 36°C) (Fig. 1a). Luminance at temperatures close to Tp was lowest in all three populations (Fig. 1a). Y values varied among populations (*F*_2, 57_=5.668, *P*<0.01), with the mean value being greatest in the ND population and smallest in the LS population (Fig. 1a; Tukey's test, all *P*<0.05). The temperature×population interaction was not a significant source of variation in luminance (*F*_12, 342_=1.43, *P*=0.150).

In both TG1 and TG2 and for each population, mean values for luminance differed between lizards measured at 24°C or 36°C and at Tp (paired-sampled *t*-test, all *P*<0.001). The differences (ΔY) in Y values differed among the three populations (TG1: *F*_2, 57_=5.782, TG2: *F*_2, 57_=7.553, both *P*<0.01). In both TG1 and TG2, mean ΔY values were smaller in the LS population than in the other two populations (Fig. 1b). From May to September, patterns of monthly changes in mean temperature and solar radiation were similar among the three populations (Fig. 1c). Cluster analysis using 19 climate variables showed that the GY and ND populations formed a clade climatically differing from the LS population (Fig. 1d).

### *De novo* assembly and annotation of unigenes

We *de novo* assembled a reference transcriptome because of the lack of genomic information on *T. septentrionalis*. A total of 487,838,568 raw reads were yielded from nine libraries. After adaptor trimming and quality filtering, 472,500,030 clean reads remained. A total of 230,477 unigenes were assembled with a mean length of 831 bp, and an N50 of 987 bp. Furthermore, ∼82% of the unigenes ranged from 300 to 1000 bp, ∼11% ranged from 1000 to 2000 bp, and ∼7% exceeded 2000 bp.

A total of 100,795 (43.7%) unigenes were annotated according to seven databases. Based on the Nr and Nt databases, 36,135 (15.7%) and 78,697 (34.1%) open reading frames were identified, respectively. In total, 26,458 (11.5%) protein-coding genes were annotated according to the Swiss-Prot database. Because of the lack of genomic information on *Takydromus*, top-hit species distribution revealed a close kinship between two lizard species, *P. vitticeps* and *Anolis carolinensis*, based on Nr annotation (Fig. S1a).

A total of 9748 unigenes were classified into 26 KOG categories; the largest group was ‘general function prediction only’ with 1625 unigenes, followed by ‘signal transduction mechanisms’ with 1568 unigenes (Fig. S1b). A total of 12,458 unigenes were mapped to 232 pathways based on KEGG Automatic Annotation Server. ‘Signal transduction’ was the most represented category, containing 1521 unigenes, followed by ‘endocrine system’ (761 unigenes), ‘immune system’ (738 unigenes), and ‘transport and catabolism’ (706 unigenes) (Fig. S1c). A total of 38,198 unigenes were classified to 1536 GO annotation; 24,042 unigenes were attributed to biological process, including 1049 GO terms; 15,712 unigenes were attributed to cellular component, including 323 terms; and 22,683 unigenes were attributed to molecular function, including 164 terms. The GO terms attributed to the greatest number of genes were ‘cellular process’, ‘function binding’, ‘metabolic process’, and ‘single-organism process’ (Fig. S1d).

### Gene expression at three temperatures

PCA including all unigenes revealed differences in gene expression among three temperature treatments, with the first two components explaining 79.3% of the variance (Fig. S2). We found that highly expressed genes were from the *COX*, *HBA*, *HBE*, *MYO*, and *KRT* families. Of the genes affecting hair color in mammals (Hoekstra, 2006), 46 homologous unigenes were detected in *T. septentrionalis*, and 40 unigenes were expressed (mean FPKM>0.3 in least two temperature treatments), with four highly expressed (mean FPKM>15) and 36 lowly expressed (mean FPKM<15) in the skin.

**Fig. 2.**
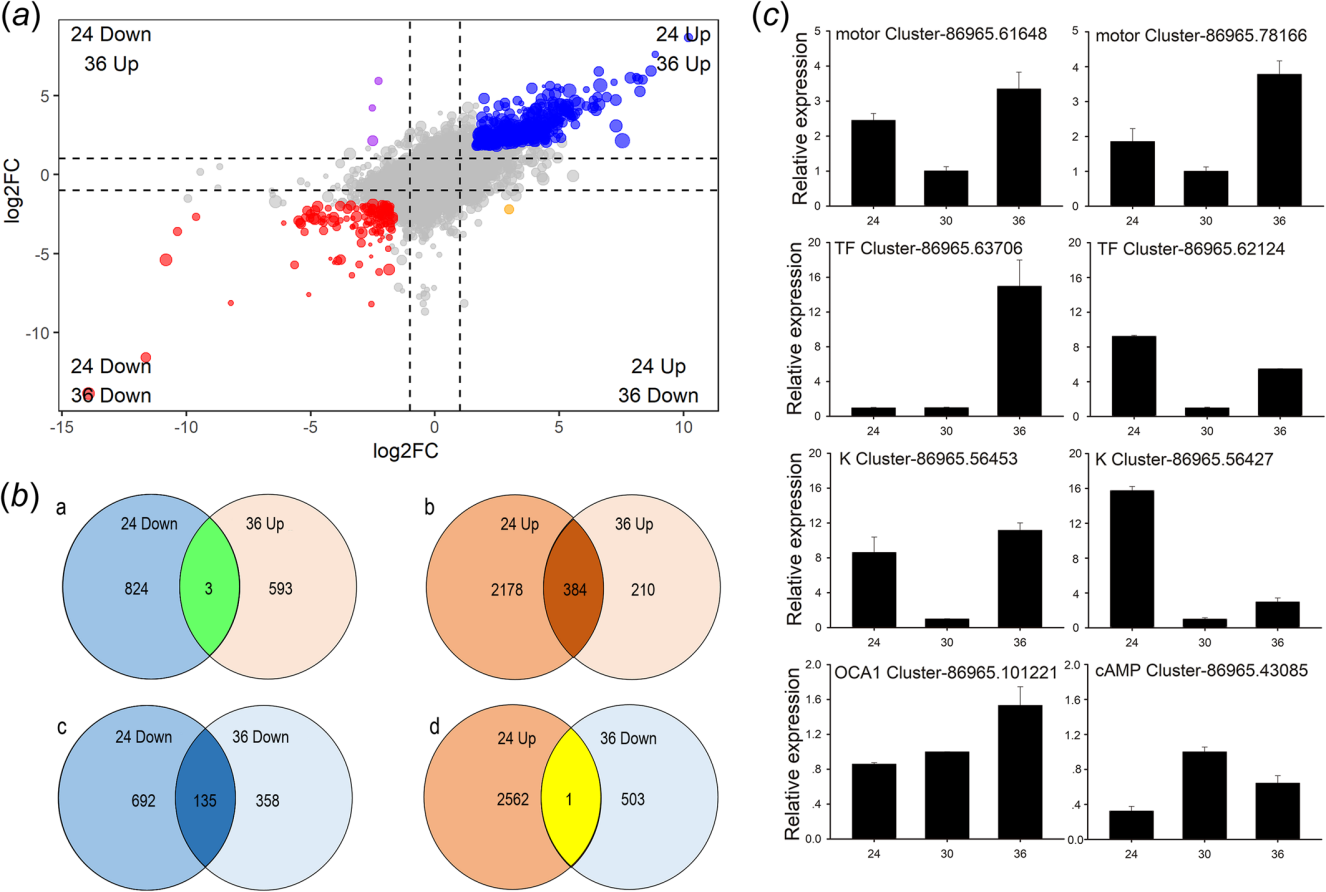
**Results of RNA-seq analysis, showing DEGs in the three temperature treatments.** (A) Volcano map of the differential expression analysis. (B) Summary of DEGs in three libraries identified through pairwise comparisons. (C) Results of qRT-PCR analysis of DEGs.

A total of 2562 upregulated and 827 downregulated unigenes were identified when the 24°C and 30°C treatments were compared (24/30); 594 upregulated and 503 downregulated unigenes were detected when the 36°C and 30°C treatments were compared (36/30); 2483 and 794 DEGs were annotated in at least one database, respectively (Fig. 2a,b; Tables S2, S3). The results of qRT-PCR were consistent with those from RNA-seq. Six DEGs (two motor protein-encoding genes, two keratin encoding genes, and two transcription factors) were upregulated at 24°C and 36°C. Expression of the downregulated cAMP-responsive element modulator (*CREM*) was suppressed at 24°C and 36°C, and the expression of *OCA1* was similar among the three treatments (Fig. 2c).

**Fig. 3.**
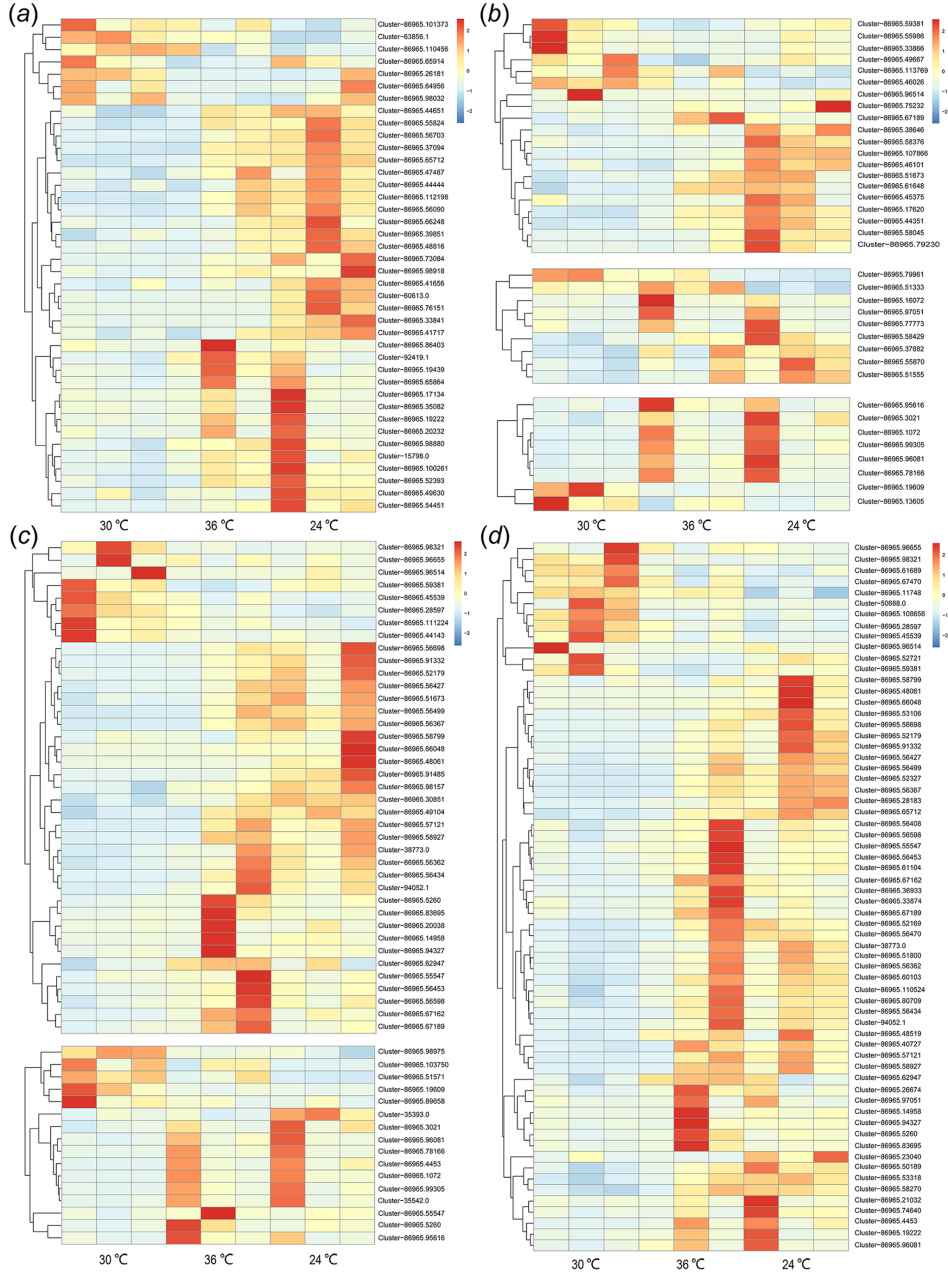
**Expression heatmaps of DEGs.** The expression pattern of genes functioned as phosphorylated kinase (A), motor proteins (including myosin, dynein and kinesin) (B), tubulin and filament (C), and cytoskeletal part (D).

Among the DEGs, 384 were co-upregulated and 135 were co-downregulated at 24°C and 36°C, while four DEGs showed an opposite expression pattern (Fig. 2b). Based on the K-means cluster analysis, the expression of numerous genes followed the same pattern at 24°C and 36°C (Fig. 3, Fig. S3). Based on the annotation of DEGs, some kinase-related (Fig. 3a) and motor protein-related (Fig. 3b) genes, and cytoskeletal genes (Fig. 3c,d), were identified, with most highly expressed at 24°C and 36°C (Fig. 3).

### Functional enrichment analysis of DEGs

#### cAMP and kinase

The expression of *CREM* was lower at 24°C and 36°C than at 30°C (Fig. 2c). Hundreds of kinase-related genes were differentially expressed, with some involved in phosphorylation (Tables S2, S3); the expression of phosphorylation-related kinase genes was thermally sensitive. Compared with the 30°C treatment, 30 unigenes were upregulated and three were downregulated at 24°C, while nine unigenes were upregulated and four were downregulated at 36°C (Fig. 3a). Six phosphorylation-related kinase genes were co-expressed at 24°C and 36°C, with all upregulated.

The GO analysis of DEGs from 24/30 and 36/30 revealed that 23 and 22 enzyme activity GO categories were enriched, respectively (Table S3). Among them, ‘the acid phosphatase activity’ was found in 24/30 and 36/30, with 12 and nine DEGs, respectively (Table 1). Nineteen and 12 DEGs in the 24°C and 36°C treatments were involved in ‘phosphatase activity’. ‘G-protein coupled receptor activity’ was also enriched in 24/30 and 36/30, including 29 and 16 DEGs, respectively, among them 24 and 11 DGEs were upregulated (Table 1).

**Table 1.**
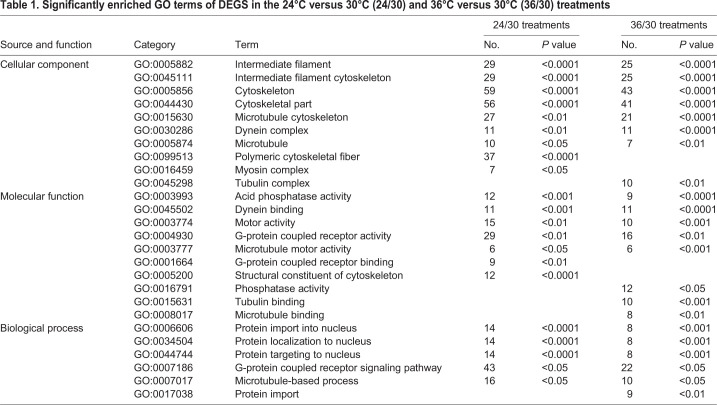
**Significantly enriched GO terms of DEGS in the 24°C versus 30°C (24/30) and 36°C versus 30°C (36/30) treatments**

#### Motor dynein hauls

Hundreds of kinesin-, dynein-, and myosin-related genes were annotated, for instance, *moy5*, *KRT1*, and *KIF*. Compared with the 30°C treatment, 30 motor protein genes (15 myosin-, eight dynein-, and seven kinesin-related genes) were differentially expressed at 24°C, and 17 genes (seven myosin-, four dynein-, and six kinesin-related genes) were differentially expressed at 36°C (Fig. 3b). More than a half of motor protein related DEGs were highly expressed at 24°C and 36°C, with 23 (12 myosin-, six dynein-, and five kinesin-related genes) upregulated at 24°C, 12 (three myosin-, four dynein-, and five kinesin-related genes) upregulated at 36°C, and nine (two myosin-, three dynein-, and four kinesin-related genes) co-upregulated at the two temperatures.

Some GO terms were associated with motor function. More than ten DEGs were contributed to ‘dynein complex’, ‘dynein binding’, and ‘motor activity’ in 24/30 and 36/30. Protein accumulation in the nucleus of cells was highly enriched, 14 and eight DEGs were annotated to ‘protein import into nucleus’, ‘protein localization to nucleus’, and ‘protein targeting to nucleus’ in 24/30 and 36/30 respectively (Table 1).

#### Cytoskeleton

Numerous tubulin and filament genes were classified, with many of them differentially expressed at different temperatures. Compared with those at 30°C, 14 tubulin-related (nine upregulated and five downregulated) and 32 filament-related (25 upregulated and seven downregulated) genes were detected at 24°C; and at 36°C ten tubulin-related (nine upregulated and one downregulated) and 25 filament-related (23 upregulated and two downregulated) genes were detected (Fig. 3c). Most of these genes were co-upregulated, including seven out of eight co-expressed tubulin-related genes and 17 out of 18 co-expressed filament-related genes (Fig. 3c; Table S2). Several other cytoskeleton related genes were differentially expressed, with most of them upregulated at 24°C and 36°C (Fig. 3d).

GO analysis of DEGs revealed many functions related to the cytoskeleton. Twenty-nine and 25 DEGs were related to ‘intermediate filament’, 27 and 21 DEGs were annotated to ‘microtubule cytoskeleton’, 56 and 41 DEGs were contributed to ‘cytoskeletal part’, and 12 and ten DEGs were related to ‘structural constituent of cytoskeleton’ in the 24°C and 36°C treatments, respectively (Tables 1, Table S3). Six DEGs were involved in ‘microtubule motor activity’ in the 24°C and 36°C treatments (Tables 1, Table S3).

### MSH and MCH

**Fig. 4.**
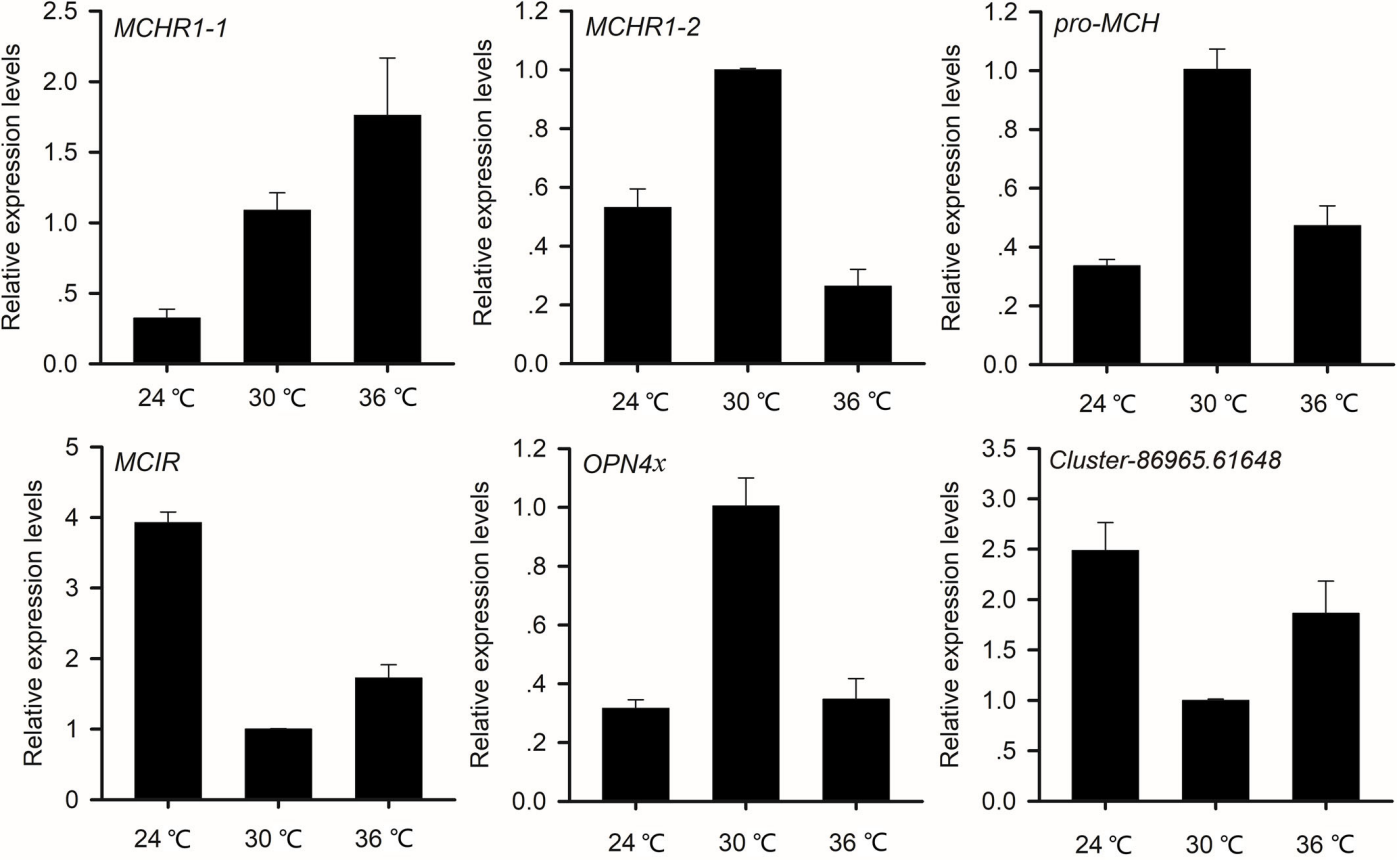
**The expression pattern of genes related to melanocyte-stimulating hormone and melanocyte-concentrating hormone at three temperatures.**

*MCHR1-1*, *MCHR1-2*, *MC1R* and the *OPN4x* orthologue were identified in our data set. QRT-PCR revealed that the expression of these genes in pituitary glands differed among the three temperature treatments. Compared with the 30°C treatment, the expression of *MC1R* was upregulated and the expressions of *MCHR1-2, pro-MCH,* and *OPN4x* were downregulated at 24°C and 36°C, and the expression of *MCHR1-1* was downregulated at 24°C and upregulated at 36°C (Fig. 4).

### KEGG analysis of DEGs

Several DEGs involved in disease-related pathways (Fig. S4), including ‘African trypanosomiasis’ and ‘amoebiasis’. In addition, at 24°C and 36°C, ‘cytokine-cytokine receptor interaction’, ‘proximal tubule bicarbonate reclamation’, ‘steroid biosynthesis’ and some metabolism-related pathways were classified.

## DISCUSSION

Body color becomes dark when pigments are dispersed throughout the cell, and light when the pigments are aggregated (Aspengren et al., 2009; Nilsson et al., 2013; Smith et al., 2016b). Kirchhoff's law of thermal radiation predicts that absorption and release of radiation energy are positively correlated (Robitaille, 2003). Accordingly, individuals with a dark color absorb or release radiant heat more rapidly than those with a light color (Seebacher, 2000). Our data show that body color is thermally sensitive in *T. septentrionalis*, and that lizards quickly change body color in response to the thermal environment around them. From the pattern of temperature-dependent color change found in *T. septentrionalis* we may conclude that pigments are more dispersed at body temperatures around Tp than at other lower or higher levels. Body temperatures vary within a range narrower than that of natural thermal fluctuations experienced by reptiles including *T. septentrionalis*, as revealed by the fact that their body temperatures are higher than ambient temperatures in the low-temperature environment and lower than ambient temperatures in the high-temperature environment (Wang and Xu, 1987; Seebacher and Shine, 2004). Thus, to reduce heat loss (due to increased release of radiation energy) at low temperatures or to avoid overheating at high temperatures, lizards should maintain body color with a relatively high luminance. Our finding that rapid color change functions as a mechanism of thermoregulation is consistent with earlier studies on *P. vitticeps* (Cadena and Tattersall, 2009; Smith et al., 2016b). Moreover, regardless of the effect of temperature, the ranges of body color variation are finite in *T. septentrionalis* from the three populations and more similar between the GY and ND populations, which are climatically more similar than other two population pairs (Fig. 1d).

The genomes have been sequenced and characterized in several species of reptiles not including *T. septentrionalis*. Transcriptase analysis may provide useful information for understanding genetic information and molecular mechanisms. Currently only a few complete genome sequences of reptiles of the suborder Sauria have been available, including *A. carolinensis* (Alföldi et al., 2011), *Shinisaurus crocodilurus* (Gao et al., 2017) and *P. vitticeps* (Georges et al., 2015). The assembled genome of sequenced lizards contains almost 30,000 protein-coding genes (Alföldi et al., 2011; Georges et al., 2015; Gao et al., 2017). We obtained 230,477 unigenes from RNA-seq, far exceeding the number of genes in the species with known genomic information. Nevertheless, this is common in transcriptome analyses of animals (Brandley et al., 2012), due to complex and abundant alternative splicing (Pan et al., 2008; Black, 2003). In humans, transcripts from 95% of multiexon genes undergo alternative splicing, and there are around 100,000 intermediate-to-high abundance alternative splicing events in major human tissues (Pan et al., 2008). In *P. vitticeps*, 19,406 protein-coding genes were obtained through genome assembly, while 595,564 contigs were obtained with Trinity for transcriptome analysis (Georges et al., 2015). Therefore, alternative splicing may be common in *T. septentrionalis.*

OAC1 and P-protein participate in pigment biosynthesis (Brilliant, 2001). In the present study, there was no significant change in the expression of these homologous genes in the three constant-temperature treatments. These results indicated that the body color change we observed in *T. septentrionalis* was not a morphological but a physiological response. High levels of cAMP facilitate protein phosphorylation and promote pigment dispersal (Nery and Castrucci, 1997; Rodionov et al., 2003). While high concentration G protein induces pigment aggregation, because G protein-coupling system inhibits adenylate cyclase activity, which can transform ATP into cAMP and result in lowered cAMP levels (White et al., 1987; Nery and Castrucci, 1997; Aspengren et al., 2009). In the present study, high expression of G protein-related genes was accompanied by low *CREM* expression. For example, at 24°C and 36°C, G protein-coupled receptor genes were highly expressed, while *CREM* expression was lower. This finding suggests that *T. septentrionalis* shares the same G protein-cAMP signaling pathway with fish (Nery and Castrucci, 1997; Nilsson et al., 2013). Pigment aggregation/dispersion is microtubule-dependent, and the concerted efforts of both microtubule and actin-based motors are required for proper pigment distribution in melanophores (Aspengren et al., 2009; Bertolesi and McFarlane, 2018). It seems likely that expression oscillations of cytoskeletal and motor-related genes at different temperatures drive pigment movement and thereby cause body color change in *T. septentrionalis*.

MSH and MCH are commonly involved in physiological color change in fish (Suzuki et al., 1995; Sugimoto, 2002; Nilsson et al., 2013). In the barfin flounder, a-MSH-rp exhibits pigment-dispersing activities *in vitro*, but not *in vivo*, because of inhibition from the nervous system (Kobayashi et al., 2010; Mizusawa et al., 2013). In our study, however, MSH may influence the pigment-dispersing *in vivo*, because the expression of *MC1R* and *OPN4x* were both changed, making it possible that the function of MSH was influenced by nervous system. In teleost fish, MCH triggers color change *in vivo* and *in vitro* (Yamanome et al., 2007; Mizusawa et al., 2013). Moreover, the findings of our study indicate that the expression of MCH-related genes was altered; therefore, we may hypothesize that MCH also affects color change *in vivo* in reptiles. In teleost fish, MCH results in the aggregation of melanin granules in melanophores (White et al., 1987; Rodionov et al., 2003; Aspengren et al., 2009; Nilsson et al., 2013). While in frogs and lizards melanophores MCH induces pigment dispersion from the center of cells (Bertolesi and McFarlane, 2018). In the present study, the expression of *MCHR1-2* and *pro-MCH* genes was significantly higher at 30°C than at 24°C and 36°C, thus resulting in pigment dispersion at 30°C. Clearly, as in frogs, MCH may have a melanin-dispersing effect in *T. septentrionalis*. In teleost fish, MCH directly binds to seven G protein-coupled transmembrane receptors, activates G proteins, and then causes pigment to aggregate rapidly (Kawauchi, 2006; Aspengren et al., 2009). However, a human and a mouse melanopsin variant (hOpn4L and mOpn4L) can activate or deactivate G-protein pathways by shifting blue/yellow light (Spoida et al., 2016). In *T. septentrionalis*, high expression of MCH-related genes was accompanied by lower expression of G protein-related genes. This finding suggests that MCH may deactivate G proteins, enhance cAMP concentration, and cause pigment dispersion in lizards.

In this study, we quantified dorsal coloration based on the RGB values in adult males of *T. septentrionalis* from three populations and exposed to different thermal conditions to test whether and, if so, how body color changes in response to the thermal environment around them. Our results showed that physiological color change was thermally sensitive in *T. septentrionalis* and had a functional role in thermoregulation achieved by altering the arrangement of pigments and thereby changing the absorption or reflection of solar radiation. Body color became increasingly light as body temperatures deviated from the level preferred by the lizard, and the thermal dependence of body color change differed among populations. From our analysis of the skin transcriptome in individuals exposed to three constant temperatures (24, 30 and 36°C) we know the following. First, G protein-coupling system inhibits adenylate cyclase activity to transform ATP into cAMP, causing the pigment to aggregate quickly. Second, MCH deactivates the G proteins and thereby initiates pigment dispersion in *T. septentrionalis*. Third, the molecular mechanism of physiological color change found in *T. septentrionalis* differs from that reported for teleost fish where the MCH activates the G proteins and thereby causes pigment aggregation. Future work could usefully investigate whether the molecular basis of the thermal dependence of physiological color change observed in *T. septentrionalis* is generalizable to lizards.

## MATERIALS AND METHODS

### Animal collection and treatment

Sixty adult males, 20 from each of three populations in Lishui (LS; 28°26′N, 119°55′E), Ningde (ND; 26°53′N, 119°39′E) and Guiyang (GY; 25°41′N, 112°34′E), were collected by noose from the field in April 2017. Lizards were brought to Nanjing, where ten from the same population were housed in each 1.2×0.6×0.4 m cage containing 50 mm-depth moist soil, grasses and pieces of clay tile. Cages holding lizards from different populations were all placed in a room where temperatures varied from 20−28°C for at least two weeks prior to testing the thermal dependence of body color change, thereby removing or minimizing the effects of potential factors other than temperature. Mealworm larvae (*Tenebrio molitor*), house crickets (*Achetus domestica*) and water were provided daily, and thermoregulatory opportunities were provided during daytime hours (08:00 to 17:00 h) using a 100 W heating light. Our experimental procedures complied with the current laws of China for the care and use of experimental animals and were approved by the Animal Research Ethics Committee of Nanjing Normal University (IACUC-200411).

### Measurement of body color change

We first used a seven temperatures × three populations repeated-measures factorial design experiment to examine the effects of temperature, population and their interaction on body color. Lizards were maintained at one of seven temperatures in a randomized sequence (24, 26, 28, 30, 32, 34 and 36°C) for 30 min to adjust body temperature at the test level. Lizards were then photographed vertically (always by Kun Guo and from the same distance) with a digital camera (Canon, Japan) using manual settings and an in-built flash unit as the light source. A black scale was included in the picture for calibration.

We then followed the procedures described above to photograph lizards on two thermal gradients (TGs) created in the same room pre-set at different temperatures. TG1 was achieved by moving cages to the room pre-set at 24°C, where a thermal gradient from 24−50°C was created by a 100 W heating light (Yang et al., 2008). TG2 was achieved by moving cages to the room pre-set at 36°C, where a thermal gradient from 12−36°C was created by a 500 ***g*** ice cube at one end of each cage. Two photos (P) were taken for each lizard: P1 at 24°C (for the TG1 treatment) or 36°C (for the TG2 treatment); P2 at the preferred body temperature (Tp ≈ 30°C), which could be achieved after a lizard was on a thermal gradient for 30 min (Yang et al., 2008).

We used MATLAB to calculate RGB values, which were transformed to luminance values (Y) using an equation of Y=16+(0.257×R+0.504×G+0.09×B) (Ahirwal et al., 2007). We used the NicheMapR script in R to obtain climate date for the three populations from global monthly hourly solar radiation and shadowless air temperature data for 2013 from the microclimate model (Kearney et al., 2014).

### RNA sequencing and transcriptome analysis

Three GY males (5.63±0.38 ***g***) were exposed to one of three temperatures (24, 30 and 36°C) for 30 min. The dorsal skin of each lizard killed by rapid freezing in liquid nitrogen was then collected and stored at −80°C for later total RNA isolation using the PureLink^®^ RNA Mini Kit (Invitrogen, USA). RNA quality detection, library preparation, and sequencing were performed according to Zhong et al. (2018). *De novo* transcriptome assembly of the clean reads was performed using Trinity 2.4.0 (Grabherr et al., 2011) with min_kmer_cov set to 3 by default. Hierarchical clustering was performed by Corset 1.05 to obtain the longest cluster sequence for subsequent analysis (Davidson and Oshlack, 2014). The final unigenes were annotated through NCBI non-redundant protein sequences (NR), euKaryotic Ortholog Groups (KOG), and Swiss-Prot, NCBI non-redundant nucleotide sequences (Nt), Protein family (Pfam), KEGG orthology database (KO) and Gene ontology (GO) as usual (Lan et al., 2018). We used edgeR 3.24.3 to analyze differentially expressed genes (DEGs) with absolute change ≥2 and FDR ≤0.05.

### Real-time quantitative PCR (qRT-PCR)

Skin and pituitary glands collected from lizards exposed to three temperatures were ground in liquid nitrogen for RNA extraction. Total RNA was isolated using TRIzol Reagent (Invitrogen, USA). Reverse transcription, qRT-PCR and data analysis were performed according to Zhong et al. (2018). Ubiquitin gene was used as the internal control. Table S1 shows primer sequences.

### Statistical analyses

We used repeated-measures analysis of variance (ANOVA) to examine the effects of temperature, population and their interaction on body color. Tukey's tests were performed to examine whether the differences between temperature treatments or populations were significant, and *P* values were corrected for multiple tests using the false discovery rate (FDR). Differences in ΔY (the absolute difference in Y between P1 and P2) among populations were tested using analysis of covariance (ANCOVA). For all the unigenes and all samples, their expression values, in terms of fragments per kilobase million, were used in principal components analysis (PCA).

## Supplementary Material

Supplementary information

## References

[BIO058503C1] Ahirwal, B., Khadtare, M. and Mehta, R. (2007). FPGA based system for color space transformation RGB to YIQ and YCbCr. *ICIAS IEEE* 1-3, 1345-1349. 10.1109/ICIAS.2007.4658603

[BIO058503C2] Alföldi, J., Di Palma, F., Grabherr, M., Williams, C., Kong, L. S., Mauceli, E., Russell, P., Lowe, C. B., Glor, R. E., Jaffe, J. D. et al. (2011). The genome of the green anole lizard and a comparative analysis with birds and mammals. *Nature* 477, 587-591. 10.1038/nature10390 21881562 PMC3184186

[BIO058503C3] Angilletta, M. J., Jr. (2001). Thermal and physiological constraints on energy assimilation in a widespread lizard. *Ecology* 82, 3044-3056. 10.1890/0012-9658

[BIO058503C4] Aspengren, S., Hedberg, D., Skold, H. N. and Wallin, M. (2009). New insights into melanosome transport in vertebrate pigment cells. *Int. Rev. Cell Mol. Biol.* 272, 245-302. 10.1016/S1937-6448(08)01606-7 19121820

[BIO058503C5] Bagnara, J. T. and Hadley, M. E. (1969). The control of bright colored pigment cells of fishes and amphibians. *Am. Zool.* 9, 465-478. 10.1093/icb/9.2.465 5362277

[BIO058503C6] Bakken, G. S. (1992). Measurement and application of operative and standard operative temperatures in ecology. *Am. Zool.* 32, 194-216. 10.1093/icb/32.2.194

[BIO058503C7] Baling, M., Stuart-Fox, D., Brunton, D. H. and Dale, J. (2016). Habitat suitability for conservation translocation: the importance of considering camouflage in cryptic species. *Biol. Conserv.* 203, 298-305. 10.1016/j.biocon.2016.10.002

[BIO058503C8] Bertolesi, G. E. and McFarlane, S. (2018). Seeing the light to change colour: an evolutionary perspective on the role of melanopsin in neuroendocrine circuits regulating light-mediated skin pigmentation. *Pigm. Cell Melanoma Res.* 31, 354-373. 10.1111/pcmr.12678 29239123

[BIO058503C9] Black, D. L. (2003). Mechanisms of alternative pre-messenger RNA splicing. *Annu. Rev. Biochem.* 72, 291-336. 10.1146/annurev.biochem.72.121801.161720 12626338

[BIO058503C10] Brandley, M. C., Young, R. L., Warren, D. L., Thompson, M. B. and Wagner, G. P. (2012). Uterine gene expression in the live-bearing lizard, *Chalcides ocellatus*, reveals convergence of squamate reptile and mammalian pregnancy mechanisms. *Genome Biol. Evol.* 4, 394-411. 10.1093/gbe/evs013 22333490 PMC3318437

[BIO058503C11] Brilliant, M. H. (2001). The mouse p (*pink-eyed dilution*) and human P genes, oculocutaneous albinism type 2 (OCA2), and melanosomal pH. *Pigm. Cell Res.* 14, 86-93. 10.1034/j.1600-0749.2001.140203.x 11310796

[BIO058503C12] Cadena, V. and Tattersall, G. J. (2009). The effect of thermal quality on the thermoregulatory behavior of the bearded dragon *Pogona vitticeps*: influences of methodological assessment. *Physiol. Biochem. Zool.* 82, 203-217. 10.1086/597483 19323642

[BIO058503C13] Clusella-Trullas, S., van Wyk, J. H. and Spotila, J. R. (2007). Thermal melanism in ectotherms. *J. Therm. Biol.* 32, 235-245. 10.1016/j.jtherbio.2007.01.013

[BIO058503C14] Davidson, N. M. and Oshlack, A. (2014). Corset: enabling differential gene expression analysis for de novo assembled transcriptomes. *Genome Biol.* 15, 410-413. 10.1186/s13059-014-0410-6 25063469 PMC4165373

[BIO058503C15] Fujii, R. (1993). Cytophysiology of fish chromatophores. *Int. Rev. Cytol.* 143, 191-255. 10.1016/S0074-7696(08)61876-8

[BIO058503C16] Gao, J., Li, Q.-Y., Wang, Z.-J., Zhou, Y., Martelli, P., Li, F., Xiong, Z.-J., Wang, J., Yang, H.-M. and Zhang, G.-J. (2017). Sequencing, de novo assembling, and annotating the genome of the endangered Chinese crocodile lizard *Shinisaurus crocodilurus*. *GigaScience* 6, 1-6. 10.1093/gigascience/gix089 PMC5569961 28595343

[BIO058503C17] Georges, A., Li, Q.-Y., Lian, J.-M., O'Meally, D., Deakin, J., Wang, Z.-J., Zhang, P., Fujita, M., Patel, H. R., Holleley, C. E. et al. (2015). High-coverage sequencing and annotated assembly of the genome of the Australian dragon lizard *Pogona vitticeps*. *GigaScience* 4, 45. 10.1186/s13742-015-0085-2 26421146 PMC4585809

[BIO058503C18] Goodson, H. V., Anderson, B. L., Warrick, H. M., Pon, L. A. and Spudich, J. A. (1996). Synthetic lethality screen identifies a novel yeast myosin I gene (*MYO5*): myosin I proteins are required for polarization of the actin cytoskeleton. *J. Cell Biol.* 133, 1277-1291. 10.1083/jcb.133.6.1277 8682864 PMC2120899

[BIO058503C19] Grabherr, M. G., Hass, B. J., Yassour, M., Levin, J. Z., Thompson, D. A., Amit, I., Adiconis, X., Fan, L., Raychowdhury, R. and Zeng, Q.-D. (2011). Full-length transcriptome assembly from RNA-seq data without a reference genome. *Nat. Biotechnol.* 29, 644-652. 10.1038/nbt.1883 21572440 PMC3571712

[BIO058503C20] Grether, G. F., Kolluru, G. R. and Nersissian, K. (2004). Individual colour patches as multicomponent signals. *Biol. Rev.* 79, 583-610. 10.1017/S1464793103006390 15366764

[BIO058503C21] Hoekstra, H. E. (2006). Genetics, development and evolution of adaptive pigmentation in vertebrates. *Heredity* 97, 222-234. 10.1038/sj.hdy.6800861 16823403

[BIO058503C22] Huey, R. B., Deutsch, C. A., Tewksbury, J. J., Vitt, L. J., Hertz, P. E., Pérez, H. J. Á. and Garland, T. Jr. (2009). Why tropical forest lizards are vulnerable to climate warming. *Proc. R. Soc. B* 276, 1939-1948. 10.1098/rspb.2008.1957 PMC2677251 19324762

[BIO058503C23] Huey, R. B., Losos, J. B. and Moritz, C. (2010). Are lizards toast? *Science* 328, 832-833. 10.1126/science.1190374 20466909

[BIO058503C24] Kawauchi, H. (2006). Functions of melanin-concentrating hormone in fish. *J. Exp. Zool. A* 305, 751-760. 10.1002/jez.a.310 16902970

[BIO058503C25] Kearney, M. R., Lsaac, A. P. and Porter, W. P. (2014). Microclim: global estimates of hourly microclimate based on long-term monthly climate averages. *Sci. Data* 1, 140006. 10.1038/sdata.2014.6 25977764 PMC4387738

[BIO058503C26] Kobayashi, Y., Tsuchiya, K., Yamanome, T., Schioth, H. B. and Takahashi, A. (2010). Differential expressions of melanocortin receptor subtypes in melanophores and xanthophores of barfin flounder. *Gen. Comp. Endocrinol.* 168, 133-142. 10.1016/j.ygcen.2010.04.017 20417636

[BIO058503C27] Lan, Y., Sun, J., Xu, T., Chen, C., Tian, R.-M., Qiu, J.-W. and Qian, P.-Y. (2018). *De novo* transcriptome assembly and positive selection analysis of an individual deep-sea fish. *BMC Genom.* 19, 394. 10.1186/s12864-018-4720-z PMC5968573 29793428

[BIO058503C28] Ligon, R. A. and McCartney, K. L. (2016). Biochemical regulation of pigment motility in vertebrate chromatophores: a review of physiological color change mechanisms. *Curr. Zool.* 62, 237-252. 10.1093/cz/zow051 29491911 PMC5804272

[BIO058503C29] Mizusawa, K., Kobayashi, Y., Yamanome, T., Saito, Y. and Takahashi, A. (2013). Interrelation between melanocyte-stimulating hormone and melanin-concentrating hormone in physiological body color change: roles emerging from barfin flounder *Verasper moseri*. *Gen. Comp. Endocrinol.* 181, 229-234. 10.1016/j.ygcen.2012.09.026 23168086

[BIO058503C30] Muri, D., Schuerch, J., Trim, N., Golay, J., Baillifard, A., El Taher, A. and Dubey, S. (2015). Thermoregulation and microhabitat choice in the polymorphic asp viper (*Vipera aspis*). *J. Therm. Biol.* 53, 107-112. 10.1016/j.jtherbio.2015.06.009 26590462

[BIO058503C31] Murillo, J. G., Rodriguez-Romero, J., Medina-Vazquez, J. A., Gonzalez-Ramirez, E. Y., Alvarez-Herrera, C. and Gadsden, H. (2020). Iridescence and thermal properties of *Urosaurus ornatus* lizard skin described by a model of coupled photonic structures. *J. Phys. Commun.* 4, 1-13. 10.1088/2399-6528/ab6510

[BIO058503C32] Nery, L. E. and Castrucci, A. M. (1997). Pigment cell signalling for physiological color change. *Comp. Biochem. Physiol. A* 118, 1135-1144. 10.1016/S0300-9629(97)00045-5 9505423

[BIO058503C33] Nilsson, S. H., Aspengren, S. and Wallin, M. (2013). Rapid color change in fish and amphibians - function, regulation, and emerging applications. *Pigm. Cell Melanoma Res.* 26, 29-38. 10.1111/pcmr.12040 23082932

[BIO058503C34] Pan, Q., Shai, O., Lee, L. J., Frey, J. and Blencowe, B. J. (2008). Deep surveying of alternative splicing complexity in the human transcriptome by high-throughput sequencing. *Nat. Genet.* 40, 1413-1415. 10.1038/ng.259 18978789

[BIO058503C35] Porter, W. P. (1967). Solar radiation through the living body walls of vertebrates with emphasis on desert reptiles. *Ecol. Monogr.* 37, 274-295. 10.2307/1942325

[BIO058503C36] Robitaille, P. (2003). On the validity of Kirchhoff's law of thermal emission. *IEEE T. Plasma Sci.* 31, 1263-1267. 10.1109/TPS.2003.820958

[BIO058503C37] Rodionov, V., Yi, J. L., Kashina, A., Oladipo, A. and Gross, S. P. (2003). Switching between microtubule- and actin-based transport systems in melanophores is controlled by cAMP levels. *Curr. Biol.* 13, 1837-1847. 10.1016/j.cub.2003.10.027 14588239

[BIO058503C38] Rogers, S. L. and Gelfand, V. I. (1988). Myosin cooperates with microtubule motors during organelle transport in melanophores. *Curr. Biol.* 8, 161-164. 10.1016/S0960-9822(98)70063-6 9443916

[BIO058503C39] Seebacher, F. (2000). Heat transfer in a microvascular network: the effect of heart rate on heating and cooling in reptiles (*Pogona barbata* and *Varanus varius*). *J. Theor. Biol.* 203, 97-109. 10.1006/jtbi.1999.1067 10704295

[BIO058503C40] Seebacher, F. and Shine, R. (2004). Evaluating thermoregulation in reptiles: the fallacy of the inappropriately applied method. *Physiol. Biochem. Zool.* 77, 688-695. 10.1086/422052 15449240

[BIO058503C41] Sinervo, B., Méndez-de-la-Cruz, F., Miles, D. B., Heulin, B., Bastiaans, E., Cruz, M. V. S., Lara-Resendiz, R., Martínez-Méndez, N., Calderón-Espinosa, M. L., Meza-Lázaro, R. N. et al. (2010). Erosion of lizard diversity by climate change and altered thermal niches. *Science* 328, 894-899. 10.1126/science.1184695 20466932

[BIO058503C42] Smith, K. R., Cadena, V., Endler, J. A., Kearney, M. R., Porter, W. P. and Stuart-Fox, D. (2016a). Color change for thermoregulation versus camouflage in free-ranging lizards. *Am. Nat.* 188, 668-678. 10.1086/688765 27860512

[BIO058503C43] Smith, K. R., Cadena, V., Endler, J. A., Porter, W. P., Kearney, M. R. and Stuart-Fox, D. (2016b). Colour change on different body regions provides thermal and signalling advantages in bearded dragon lizards. *Proc. R. Soc. B* 283, 626-634. 10.1098/rspb.2016.0626

[BIO058503C44] Spoida, K., Eickelbeck, D., Karapinar, R., Eckhardt, T., Mark, M. D., Jancke, D., Ehinger, B. V., Konig, P., Dalkara, D., Herlitze, S. et al. (2016). Melanopsin variants as intrinsic optogenetic on and off switches for transient versus sustained activation of G protein pathways. *Curr. Biol.* 26, 1206-1212. 10.1016/j.cub.2016.03.007 27068418

[BIO058503C45] Stuart-Fox, D. and Moussalli, A. (2009). Camouflage, communication and thermoregulation: lessons from colour changing organisms. *Phil. Trans. R. Soc. B* 364, 463-470. 10.1098/rstb.2008.0254 19000973 PMC2674084

[BIO058503C46] Sugimoto, M. (2002). Morphological color changes in fish: regulation of pigment cell density and morphology. *Microsc. Res. Techniq.* 58, 496-503. 10.1002/jemt.10168 12242707

[BIO058503C47] Summers, C. H. and Greenberg, N. (1994). Somatic correlates of adrenergic activity during aggression in the lizard, *Anolis carolinensis*. *Horm. Behav.* 28, 29-40. 10.1006/hbeh.1994.1003 8034280

[BIO058503C48] Suzuki, M., Narnaware, Y. K., Baker, B. I. and Levy, A. (1995). Influence of environmental colour and diurnal phase on *MCH* gene expression in the trout. *J. Neuroendocrinol.* 7, 319-328. 10.1111/j.1365-2826.1995.tb00764.x 7647775

[BIO058503C49] Wang, P.-C. and Xu, H.-F. (1987). The influence of ambient temperature on body temperature and heat energy metabolism of *Takydromus septentrionalis*. *Acta Herpetol. Sin.* 6, 10-15.

[BIO058503C50] Wang, Z., Ma, L., Shao, M. and Ji, X. (2017). Are viviparous lizards more vulnerable to climate warming because they have evolved reduced body temperature and heat tolerance? *Oecologia* 159, 19-31. 10.1007/s00442-017-3979-0 29018950

[BIO058503C51] White, B. H., Sekura, R. D. and Rollag, M. D. (1987). Pertussis toxin blocks melatonin-induced pigment aggregation in *Xenopus* dermal melanophores. *J. Comp. Physiol. B* 157, 153-159. 10.1007/BF00692359 3571570

[BIO058503C52] Yamanome, T., Chiba, H. and Takahashi, A. (2007). Melanocyte-stimulating hormone facilitates hypermelanosis on the non-eyed side of the barfin flounder, a pleuronectiform fish. *Aquaculture* 270, 505-511. 10.1016/j.aquaculture.2007.05.037

[BIO058503C53] Yang, J., Sun, Y.-Y., An, H. and Ji, X. (2008). Northern grass lizards (*Takydromus septentrionalis*) from different populations do not differ in thermal preference and thermal tolerance when acclimated under identical thermal conditions. *J. Comp. Physiol. B* 178, 343-349. 10.1007/s00360-007-0227-7 18071715

[BIO058503C54] Zhong, J., Peng, Z., Peng, Q.-L., Cai, Q.-Q., Peng, W.-L., Chen, M. and Yao, J.-L. (2018). Regulation of plant height in rice by the Polycomb group genes *OsEMF2b*, *OsFIE2* and *OsCLF*. *Plant Sci.* 267, 157-167. 10.1016/j.plantsci.2017.11.007 29362094

