# Supplementary material for: The thermal dependence and molecular basis of physiological color change in Takydromus septentrionalis (Lacertidae)
Source: Biol Open. 2021 Mar 26;10(3):bio058503. doi: 10.1242/bio.058503 (PMC8015239; doi:10.1242/bio.058503)
Supplement: Supplementary information [file biolopen-10-058503-s1.pdf]

Biology Open (2021): doi:10.1242/bio.058503: Supplementary information

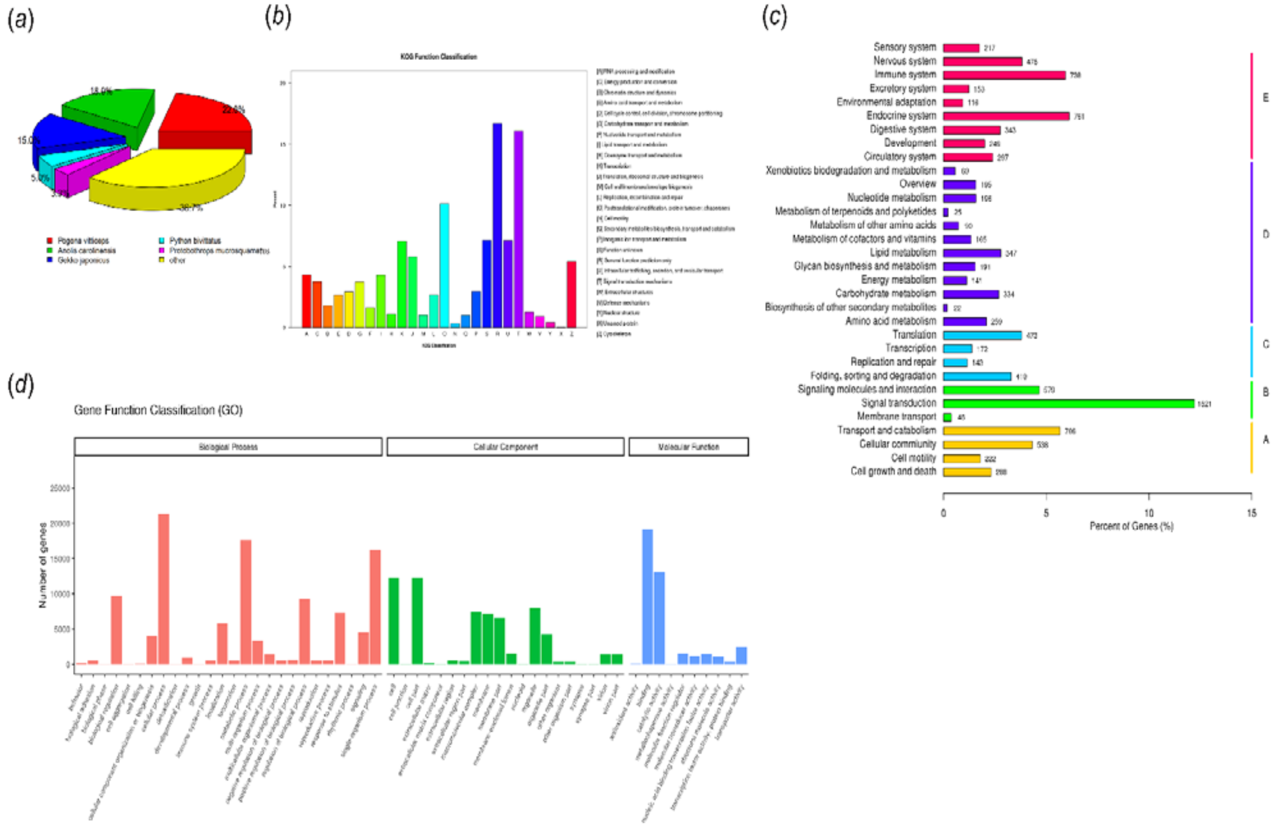

Figure S1**.** Gene annotation and function classification. A: Homology searches of Illumina sequences against the Nr database, and the species distribution of unigenes. B: KOG functional categories in the *T. septentrionalis* transcriptome. The x-axis represented the category name, and the y-axis represented the percent of unigenes. C: KEGG functional categories. The y-axis is the name of KEGG metabolic pathway, and the x-axis is the number of genes annotated to the pathway and the percent of unigenes. According to the involved KEGG metabolic pathway, genes can be divided into five branches, cellular processes (A), environmental information processing (B), genetic information processing (C), metabolism and organic systems (D), and organismal systems (E). D: GO classifications. The x-axis is the GO term at the next level of the three categories of GO, and the y-axis is the number of genes annotated to the term or subterm.

Biology Open • Supplementary information

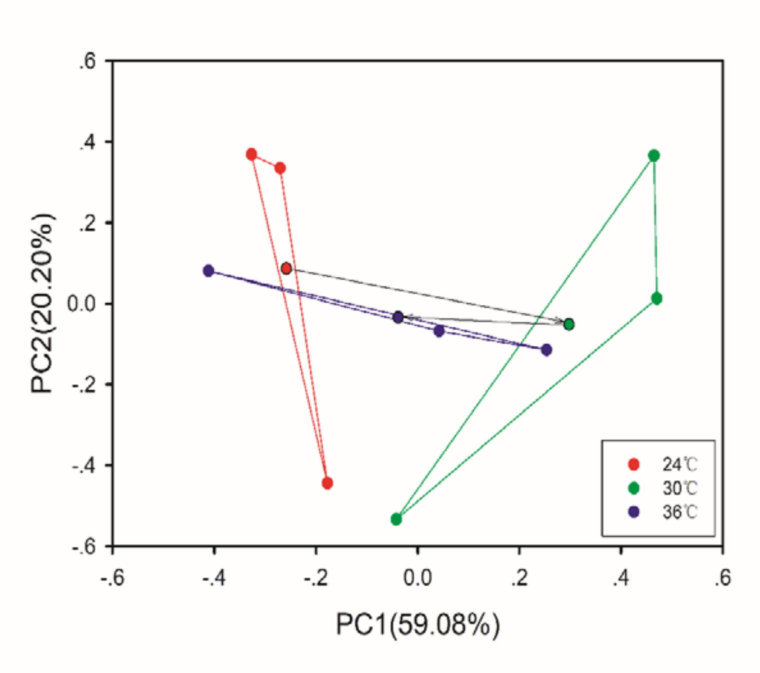

Figure S2. PCA analyses of gene expression in the three temperature treatments.

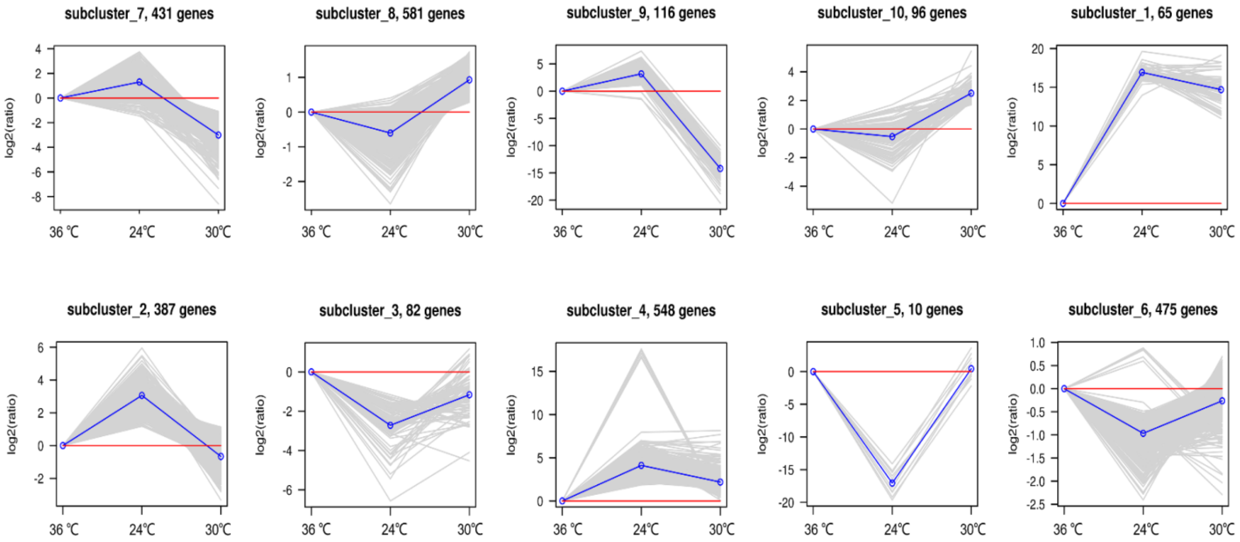

Figure S3. K-means clustering analysis of differential gene expression.

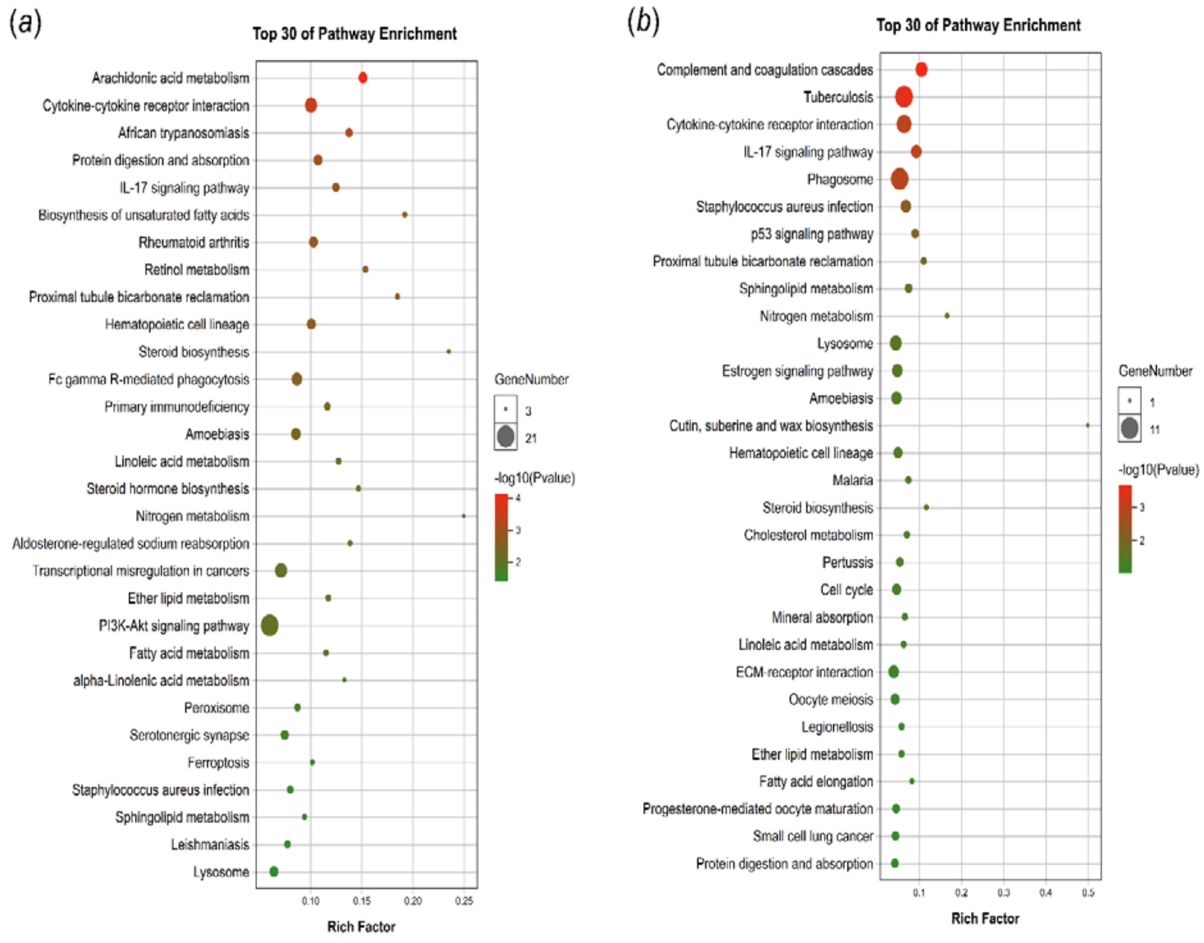

Figure S4. Results of KEGG analysis of DEGs at 24 °C (a) and 36 °C (b) compared with those at 30 °C.

Biology Open • Supplementary information

Table S1 Primers used for qRT-PCR in this study

Click here to Download Table S1

Table S2 Details of DEGs in the 24/30 °C treatments

Click here to Download Table S2

Table S3 Details of DEGs in the 36/30 °C treatments

Click here to Download Table S3
